# New York Odontological Society

**Published:** 1891-05

**Authors:** 

**Affiliations:** New York Odontological Society


### Reports of Society Meetings.

---

### NEW YORK ODONTOLOGICAL SOCIETY.

The New York Odontological Society held its regular meeting at the Academy of Medicine, No. 17 West Forty-third Street, New York City, on Tuesday evening, February 17, 1891.

The President, Dr. William H. Dwinelle, in the chair.

### INCIDENTS OF OFFICE PRACTICE AND CASUAL COMMUNICATIONS.

*The President.*—Under this head, we have a communication from an associate member of this society, William Herbert Rollins, M.D., D.M.D., of Boston, which the secretary will please read.

Dr. George Wilson, the Corresponding Secretary, read the paper from Dr. Rollins, entitled "Office Notes."

(For Dr. Rollins's paper, see page 292.)

*The President.*—If no one is ready to discuss Dr. Rollins's paper I will introduce the speaker of the evening, Professor J. S. Wight. The subject of the essay is "Work," with which we are all familiar.

(For Professor Wight's paper, see page 284.)

*The President.*—Gentlemen, we have all listened with pleasure to this address upon a subject which interests us so deeply, and I now invite discussion upon it. If we should call upon those who have most distinguished themselves in work, according to their age, I suppose we would call upon our worthy friend, Dr. Atkinson. I do not know any one who could respond better than he.

*Dr. W. H. Atkinson.*—It is not my fault that I am old; it is my fortune that I am young in aspiration for truth and excellence; not for myself, but, as the closing sentence of the paper we have listened to indicates, the work worthy to be done is not for ourselves, but for others.

Where do we stand, as compared to the work that has been commended so highly to us, that was initiated by the men called "the fathers of medicine?" We stand on vantage ground to them in everything respecting the history of medicine; but we do not stand one jot ahead of them in the position of the attainment of any knowledge, for they, as the Scriptures tell us, got all they have through the inspiration of the Almighty, that giveth man understanding. If what we call understanding were legitimate understanding, and endued us with the power to bring to pass the good we aspire to, then indeed might we seek for a syllabus that should be capable of being taught with mathematical certainty, so as to know when we went against it or when we coincided with it. Any one who facilitates the return to health from illness is a healer, to the extent of the work that is done in that regard. My nature rises up always, I do not know but wickedly, against the assumption that there is higher work than handicraft; that there is higher work than the cultivation of the soil; that there can be a more legitimate exercise than the preparation of food, raiment, and shelter; and I do hate the division that says there is a difference between workers at all, and that it is legitimate to make a difference, and call certain workers professional men, and other workers mechanics, etc.

There is a mechanism in everything that is executed; if we knew how beautiful the nutrition of the gray matter of the brain appears under the impact of the desire to know, we might say that we understand something about mental activity, something about moral relation, and about what makes us happy when good results follow our efforts, and miserable when we have inflicted wretchedness on our fellows. I suppose you think I am a preacher, too. It is good to preach when you have anything to say; but how shall they be taught without a preacher, and how shall they preach unless sent? I was not sent; but I was called by the chair to speak, and the subject is so great, so manifold, and so involved that I tremble before it whenever I think of speaking about that which is normal and that which is abnormal, and how they can be regained after they have been lost.

All that we do is involved to such an extent as to make it necessary that we do not teach by preaching alone, but by exhibition of the works that we do, and until we curtail didactic instruction we will not make much progress. Does that say that we have had no progress? The faces in this room show we have had progress. How did we get it? Not by following text, but by showing what we could do, each to the other, and inquiring each of the other how he did certain work; and thus have we gone on, until this blessed day of light, that enables the humble dentists to do more for the comfort of age than all the other professions that have preceded it, —keeping their digestory organs in good condition, by intelligently understanding the laws of life, and, as Dr. Holmes so beautifully said, "it has annihilated age."

*The President.*—I hope this discussion will continue. Dr. Jarvie, we would like to hear from you.

*Dr. William Jarvie.*—I do not know that there is anything I can say to add at all to the interest of the meeting. The paper is of such a character that I do not think any one can antagonize the statements it contains. I am very much impressed with the title of the paper,—"Work,"—the desirability and necessity of work. It is by work that we progress; it is by work that we grow; it is because of work that we, as a profession, are not where we were twenty years ago; and it will be by the constant application of that word that this profession will continue in the advanced position that it has been assuming during the past many years.

*The President.*—Dr. Davenport, I am sure you have thought a great deal on this subject; we would like to hear some of the products of your thoughts and conclusions.

*Dr. S. E. Davenport.*—I am afraid, Mr. President, my thoughts have been directed more to the latter part of the essay, to the extremely practical portions, with which I am greatly in sympathy, where we are urged not only to do our work for the good that may come from it, without special thought to the remuneration to be received, but also that we shall not place an unusual or a higher value upon our services, even though we may have been given a certain ability, perhaps above our neighbors, in improving certain methods or appliances for the relief of distress.

The essayist has perhaps not been able to follow the drift of the question of patents as it has come before this Society in the past year and a half, but I feel sure that the missionary work in both word and deed which has been done by several prominent members of our specialty has enabled us to progress largely in the right direction. I know there are several members of this Society who in the past few years have made valuable contributions to the art of the profession, and who for reasons they thought to be good at the time did patent them. I believe they have since made up their minds that the reasons governing that action were not sufficient, and that to-day the products of their minds are given freely to the profession.

I feel under obligations to the essayist for admonishing us in the very pleasing way he has, and I think it deserved, for I am afraid we think too much of fees, and particularly as the term fee has become so common. We are apt to feel that services rendered, no matter of what character, must be followed by the receipt of a fee for the time expended, reasoning with ourselves that our time is just so valuable. That may in a measure be right, but surely the success of the operation, the character of the services rendered, and the ability of the patient to pay should have more to do with the size of the fee than any iron-clad rule which we may make.

*The President.*—We would all like to hear from Dr. Howe on the subject.

*Dr. J. Morgan Howe.*—Mr. President, I have been very greatly pleased with Professor Wight's essay, but I will merely discuss for a moment that portion of it which makes the greatest impression on my mind, and it is the thought he has presented of the dignity and importance, the inherent nobility, of work,—of the heart, of the head, and of the hand. More than once during the past few years I have thought of the future of dentistry. It has occurred to me that the young men who are coming forward to practise our art may have less interest in the work to be done for its own sake, and for the sake of the good it would do those for whom it was rendered, than for the remuneration that was expected to be received. It seems to me that the thoughts of the essayist which are directed to this point are of great importance not only to those of us who have heard them, and those who will read them in the larger circulation of our transactions, but also to the young and coming generation of dentists who will soon take our place. Unless a sentiment of pride and interest in the work which they are to do for the sake of the good it will accomplish, and of pride in the perfection to which they can bring that work aside from the fee, can be cultivated to a greater extent than I fear it has been, the work of the coming dentist will not take rank with that of our honored president, and some others whom we might mention.

Of course, all who have heard me discuss the question of patents know that I heartily endorse what I understand to be the position of our essayist on that point.

*The President.*—This subject is increasing with interest as we discuss it. I knew it would. We would like to hear from Dr. Perry.

*Dr. S. G. Perry.*—I can only commend the spirit of the paper, as we know it is our habit to have papers that are specially directed to the discussion of special subjects, and it is interesting to have a paper of this kind in which occur so often the well-known, old-fashioned terms of goodness, honor, virtue, justice, altruism, and terms of that sort, that after all should be kept well before our minds in these modern days of work. In the haste of the materialistic and industrial age in which we live it may not be strange that they sometimes are forgotten.

I do not know whether I can altogether agree with Dr. Howe as to the coming members of the profession. There is this to be remembered: that there are but a few mountain peaks, and there never will be many again. There cannot be in the nature of things, for the valleys are rising. It is a very hopeful sign, it seems to me, that the younger men are working and lifting the valleys, as one might say, though they are working slowly, and many are not yet heard from. I have great faith in the coming generation of dentists. I often see work from distant towns from the hands of men whom I have never heard of before, that I look upon with surprise. I am sure throughout the country there is a higher standard than ever existed before, and if it is an industrial age in which we live, and one in which it would seem that morals are not cultivated, and in which luxury abounds, yet, on the other hand, we hear of the bad and not of the good. I believe with Emerson, in his last essay on the "Republic," that things are bettering and that that is the inevitable tendency. It certainly applies to our profession, and no one can notice it so well as the older members of the profession. I can sympathize with Dr. Atkinson when he lays so much stress on the changes that have occurred in his time, and I listen with much interest to his hope in the younger men, for my opinion is that the dental profession has more before it than back of it.

As to the subject of patents, I think there can be no doubt as to the future of that question. I do not for a moment believe that the dental profession will tolerate in the future the taking of patents, unless it be for the purpose of protecting devices from those who would patent them and monopolize them. I am not sure but that to take a patent for the purpose of protecting it, so that it can be presented to the profession, may be wise sometimes, but that the patentee should profit by it, will not, in the course of a few years, be tolerated by the profession.

I have had something to do with it, and I regret nothing in my life so much as that; but I did it with such light as I had at the time, and with little profit to myself.

The subject of fees has been spoken of. I think fees are individual. I do not believe that any just law can be laid down that will apply to all alike. I have never charged very high fees, because to this day I have not had a very high opinion of my work. It constantly falls below my ideal, and I have never been able to bring myself to the point of thinking that it is worth so very much, and if it is not worth so much, then I cannot charge for it. I do not, however, advocate or defend low fees, for it is evident that to charge a higher fee is very likely to lead a dentist to appreciate more fully the importance and value of his work, and perhaps lead him unconsciously to place the real value on the organs upon which he works. It is well to let the mind dwell on the fact that the teeth upon which we work are of immense value, and that everything we do on them is worthy of the strictest care and closest attention to all minute details,—that it is not boys' play, nor work for children. But it is work worthy of the best efforts of the best men that ever lived.

*The President.*—Dr. Bogue, we would like to hear from you. We know you are well acquainted with the subject of the essay.

*Dr. E. A. Bogue.*—Mr. President, I do not quite know which of several meanings to put on that little friendly remark. If it means that I am well acquainted with digging, yes. As I listened to the essayist, I could not help agreeing with his views with all my heart. Neither could I help thinking to myself, how little, after all, one in a kindred calling can see into our work. When he came to speak of the question of fees, I thought of the rule which has been mine through life, and which, I believe, is supported by most of the gentlemen present. This rule is to ask myself, first, "Can I afford to take this patient?" If the patient is accepted, the question of fee is dropped, and then the question comes, "What is the best thing that can be done for this patient?" And with that thought in mind, I have been proud to see the manifest advance which has been made in our handicraft; for honest work shows the thought that is behind it. No longer ago than last evening I heard a lecture on Egypt, and the lecturer said that people who did such work in their architecture could not have been dishonest people; they must have been true to the interests of life; they must have had truth as a basis for their work.

Let me show you an example of that to-day. A few months ago I wanted a sixth-year molar to transplant, and I sent to all the extractors I knew anything about. Up to now I have failed to procure it, but I did receive a letter the other day from a gentleman, saying that a little while ago I could have gotten all the good sixth-year molars I wanted, but now it is impossible to obtain them. My delight at not being able to get what I wanted may be imagined.

In regard to this question of patents, which causes some of our friends to hang their heads a little, I hope they will also remember that we are vastly different from what we were a few years ago.

Illustrating that, perhaps I may allude to a little incident. At a dinner in France, given to one of the officers of this Society, the president of the French society made a few remarks, and alluded pleasantly to the Odontological Society, and what we had done for them. In my answer I said that thirteen years ago graduates of the French college of medicine had refused to let their own apprentices and associates stand by the operating-chair to see their operations, because they wished to keep them secret. For my part, I believed I had been into the most secret recesses of many of the best dental offices in France, with the full knowledge and consent of the doctors after informing them that I should publish any specially good thing that I might find. The answer came very quickly, "Yes, you have been to see our processes, but only because you and your friends permit us to see yours." The same illiberality which I saw thirteen years ago in France Dr. Straw told me existed here about twice that length of time ago. I think gentlemen in the room may certify for or against it. We are developing; we are growing; and while there is much room to develop, I hope the good advice we have received this evening, and in which we can all fully agree, will not be wasted.

*Dr. Perry.*—A very eminent man, some time since, expressed to me the thought that as art loses its repose, it enters upon its period of decadence. I am inclined to think that this is a fact of universal application. Of course, the idea he wished to express was that the greatest works of art conveyed the thought of repose, and that if that restfulness and repose were not conveyed, that they were on the downward path.

I see one fear, one need, and that is in this hurried age we do not have that repose which is necessary for perfect work. If that could be more fully realized, and if we could never be induced to hasten or hurry,—and I speak with feeling on this subject, for my life has been too busy,—we should attain better results with less wear and tear of mind and body. It seems to me that one of the deductions that can be fairly drawn from the paper of the evening is that restfulness and repose are only other words for truthfulness in work.

*Dr. Lord.*—I think we have all been interested in the paper that has been read to us, and we will all leave this place feeling that we may be the better for it. We cannot but be impressed with the most delightful, earnest spirit of the essayist, and I move you, Mr. President, that we pass a most hearty vote of thanks to the essayist for his very interesting and suggestive paper.

Motion seconded and carried.

*Dr. Atkinson.*—I have a word to say about this idea of repose when thoroughly understood. I agree with Dr. Perry, but I have noticed that when we were doing our best work, there was an enthusiasm that came over us, which gave us illumination and ability before unknown and unattained, so that if we are in the spirit of comprehending the principles involved, and executing the work in hand, we will make progress against that kind of stillness or quiet of repose, as generally understood. It is being swallowed up, so that the individual no longer stands out as a worker, but he is part and parcel of the work, just as the drops of the sea are of the infinite ocean, without distinctness of manifestation.

Personally speaking, I feel that I do not know the lapse of time, or anything as to the consequences that in my best mood invoked my most enthusiastic efforts in doing the work. But that is progress. It is made for me.

Adjourned.

S. E. Davenport, D.D.S., M.D.S.,
*Editor New York Odontological Society.*

